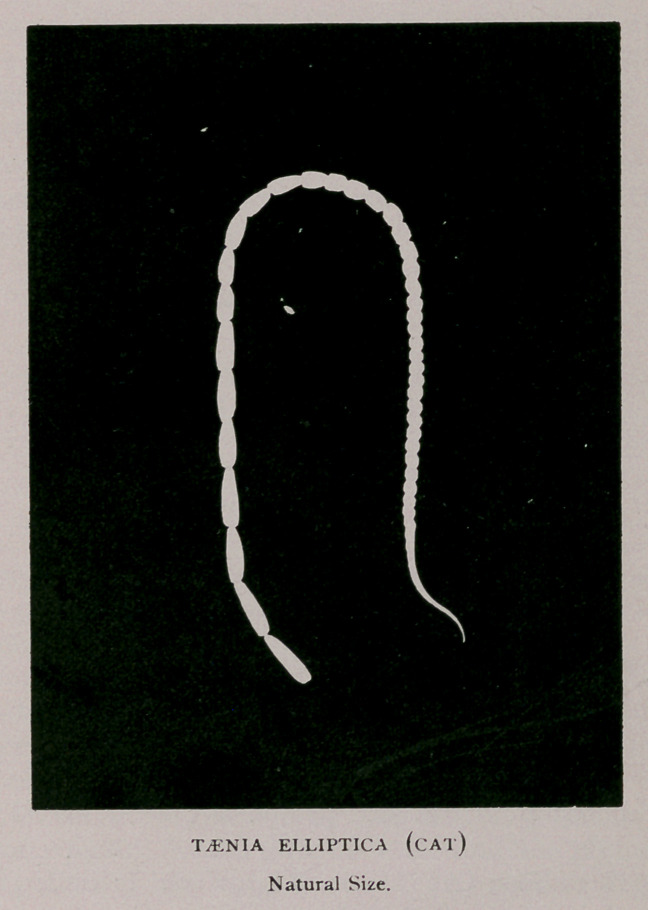# Note on Tænia Elliptica (Cat)

**Published:** 1888-04

**Authors:** William S. Gottheil

**Affiliations:** New York


### Art. IX.—NOTE ON TÆNIA ELLIPTICA (CAT).

BY WILLIAM S. GOTTHEIL, M. D.,

*New York.*

Not less than five varieties of cestode parasite affect the common cat. They are:

1. *Bothriocephalus decipiens*—a very rare form.
2. *Tœnia litterata*—especially prevalent in Iceland and Northern Europe.
3. *Tœnia lineata*—found only in the wild cat.
4. *Tœnia crassicallis*—fairly common. The intermediate hosts are known to be rats and mice, in whose livers the encepted larvæ of the parasite are not uncommonly found, where they have been described as *cysticercus fasciolaris.*
5. *Tœnia elliptica*, of which we give a photograph of life size.

Tænia elliptica is closely related to tænia cucumerina of he dog. Nevertheless, there is sufficient difference between them to warrant one regarding them as separate varieties, at least, of one species. A comparison of the accompanying illustration with Cobbold's drawing makes this quite evident. The so-called neck, which here measures only a line, is there some two inches long; and the

TÆNIA ELLIPTICA (CAT)

Natural Size.

shape and size of the segments, and of the head, is entirely different.

The entire length of the longest specimen in my possession is only 4½ inches, and the mature and free proglottides were no larger than the terminal one in the specimen here represented. In the last segment one lateral sexual orifice is open, and its contents have already been expelled. Mature segments are 3-10 x 1-10 inch, flattened, elliptical, and not overlapping. Reproductive organs double and laterally placed. The papillæ at which they open can be distinctly seen in some places.

The intermediate host in this case is said to be the cat louse, though no pediculi were found upon the animal from whom this specimen was taken.

## Figures and Tables

**Figure f1:**